# Thirsting to Death

**Published:** 1864-10

**Authors:** 


### THIRSTING TO DEATH.

It ought not to be forgotten by any one liable to shipwreck that thirst is quenched by soaking the clothing in salt water twice a day, or even oftner, and allowing them to dry upon the person. A noble and humane old sea captain, Kennedy, published this statement more than a hundred years ago ; yet it is very doubtful if two persons out of any company, taken promiscuously, are aware of so important a practical fact, to which the generous captain attributed the preservation of his own life and of six other persons. If sea water is drank, the salty portions of it are absorbed into the blood and fires it with a new and more raging thirst and a fierce delirium soon sets in. It would seem that the system imbibes the water, but excludes all the other constituents. It is known that wading in common water quenches thirst with great rapidity. Persons while working in water seldom become thirsty. And it is further interesting to know, that however soaking wet the garment may become from rain or otherwise, it is impossible for the person to take cold if the precaution is taken to keep in motion with sufficient activity to keep off the feeling of chilliness, until the clothing is perfectly dried or facilities are afforded for a change ; but in changing the garments after a wetting, it is always safest and best, as an additional safeguard against taking cold, to drink a cup or two of some hot beverage before beginning to undress.

